# Multi-stage Friend murine erythroleukemia: molecular insights into oncogenic cooperation

**DOI:** 10.1186/1742-4690-5-99

**Published:** 2008-11-04

**Authors:** Françoise Moreau-Gachelin

**Affiliations:** 1 Inserm U830, 26 rue d'Ulm, 75248 Paris cedex 05, France; 2 Institut Curie, 26 rue d'Ulm, 75248 Paris cedex 05, France

## Abstract

The Friend virus SFFV (Spleen Focus Forming Virus) provokes an acute erythroblastosis in susceptible strains of mice that progresses to overt erythroleukemia by a multi-step process. For virologists, the Friend virus-induced disease has provided deep insights into the host mechanisms influencing susceptibility to retroviral infection and viremia. These insights have contributed to the understanding of HIV and other human retroviral infections. For cell biologists and oncologists, this leukemia has been a powerful experimental model to identify critical oncogenes involved in a multi-stage process, to understand the contribution of host genes to cancer development, and to investigate the mechanisms leading to cell growth autonomy. This model also provided an example of oncogenic reversion since Friend tumor cells can reinitiate their erythroid differentiation program when exposed *in vitro *to some chemical inducers. This review highlights recent findings demonstrating that the leukemic progression depends on the cooperation of at least two oncogenic events, one interfering with differentiation and one conferring a proliferative advantage. The Friend model of leukemia progression recapitulates the two phases of human acute myeloid leukemia (AML). Coupling of insights from studies on the Friend erythroleukemia with knowledge on AML might allow a better understanding of the molecular mechanisms involved in the evolution of leukemia in mice and men.

## Review

Friend retroviruses and Friend disease have provided powerful experimental tools in the fields of virology and oncology. During the nineteen-seventies, they have been of great importance in understanding the mechanisms of resistance of mice to retroviral infection, leading to the identification of several host genes that affect Friend virus replication in target cells and control host immune response to the viral antigens [1,2]. Recent advances made in primates retroviral restriction activities have given major relevance to the previously accrued murine data[3,4]. For example, the retroviral restriction factor Fv1 (Friend virus susceptibility factor 1) [5-8] provides resistance to infection by particular murine leukemia viruses, and the Ref1/Lv1/TRIM5 factor mediates resistance to diverse retroviruses in primates, including humans [9-11]. These mechanisms of resistance target a common capsid molecular determinant [12,13]. Remarkably, the murine gene *Rfv3 *(Recovery from Friend virus 3) which influences Friend viremia [14] and retroviral neutralizing antibody responses [15] encodes APOBEC3 known otherwise as a powerful antiretroviral factor that restricts retroviral virions of cognate origin [16-19]. Moreover, the erythroleukemia induced in mice by Friend retroviruses represent experimental neoplasms that have been valuable for characterizing the multi-stage progression of leukemia, for identifying oncogenes or tumor suppressor genes involved during this process, for understanding the contribution of host genes to cancer, and for investigating the mechanisms leading to cell growth autonomy. The purpose of this review is to highlight the important insights that have arisen from the studies on the Friend disease and to discuss the molecular events involved in the outgrowth of preleukemic cells and their progression to malignancy.

### Friend diseases

**Figure 1:**
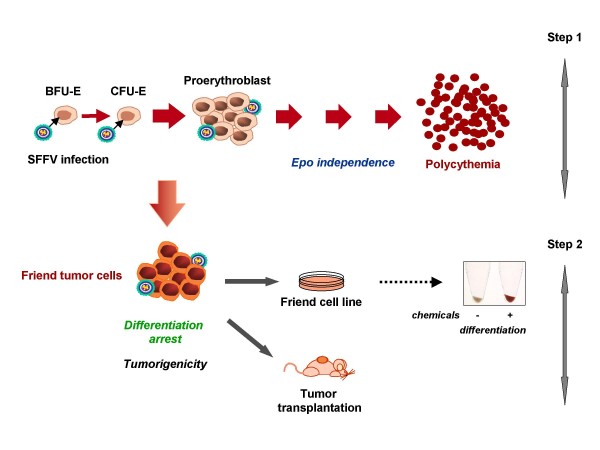
**Friend erythroleukemia is a two-step disease**. The target cell for SFFV is a late BFU-E or a CFU-E. SFFV infection induces the proliferation of proerythroblastic cells that differentiate into erythrocytes in the absence of Epo, in the case of FLV-P infection. The second step is characterized by the outgrowth of Friend tumor cells that can be serially transplanted into mice and cultured as permanent cell lines without growth factor. The tumor cells are blocked in differentiation, but can be induced to differentiate along the erythroid pathway by chemical inducers. A pellet of red blood cells derived from a Friend tumor cell line treated for 5 days with DMSO is shown in the box.

Friend acute erythroleukemias are caused in susceptible mice by various strains of Friend leukemia viruses (FLV). The description of the initial isolate took place 50 years ago when the American virologist Charlotte Friend observed that newborn mice inoculated with Ehrlich murine carcinoma cells developed an acute erythroblastosis that could be subsequently transmitted by cell-free extracts prepared from the spleens of diseased mice [20]. The early description of the disease indicated that the original isolate (termed anemia-inducing Friend virus or FLV-A) caused an anemia mainly due to hemodilution [21]. Since the original report by C. Friend, a number of other isolates capable of inducing a disease spectrum comparable to FLV-A have been identified. Among these, the Rauscher isolate was biologically similar to FLV-A [22]. A few years later, derivatives were obtained. In contrast to FLV-A causing an anemia, the derivatives increased the levels of erythrocytes in the peripheral blood (polycythemia) and was termed polycythemia-inducing Friend virus or FLV-P [23]. Adult or newborn mice inoculated with either FLV-A or FLV-P develop a disease with no latency. Both virus isolates induce an erythroblastosis that rapidly progresses to acute transformation (Figure 1). The early stage is characterized by a rapid increase in the number of proerythroblastic cells leading to massive hepatosplenomegaly that may be fatal by accidental spleen rupture. These proerythroblasts die *in situ *or differentiate into abnormal erythrocytes either in the absence of erythropoietin (Epo), in the case of FLV-P, or with a hypersensitivity to Epo, in the case of FLV-A[24-27]. The target cell in which both FLV-P and FLV-A express their pathogenic effect is an Epo-responsive progenitor cell that was identified as a late BFU-E or a CFU-E [25,27,28]. Although Friend virus-infected erythroid progenitors dramatically increase in number, they retain limited proliferation capacity and are not tumorigenic *in vivo*. Within two to three weeks after virus inoculation, a clonal population of proerythroblastic cells blocked in their differentiation program first emerges in the spleen and then invades the animal [29-31]. These leukemic cells have extensive proliferative capacity, the ability to be serially transplantable *in vivo*, and the capacity to eventually establish permanent cell lines *in vitro*. Cell lines established in culture were called Friend tumor cells. Of note, the erythroid differentiation program can be reinitiated in Friend tumor cells when exposed *in vitro *to some chemical inducers like dimethyl sulfoxyde, hexamethylene bisacetamide, butyric acid or hemin [32]. This contrasts importantly with B-lymphocyte or T-lymphocyte transformation by Bovine Leukemia Virus (BLV) or Human T-cell Leukemia Virus (HTLV)[33] which are phenotypically irreversible. Thus, the Friend tumor cells have provided a useful *in vitro *model for studying the molecular processes of erythroid terminal differentiation.

### Friend viruses

Both FLV-A and FLV-P isolates contain at least two viral components: a replication-competent Friend murine leukemia virus (F-MuLV) and a replication-defective spleen focus forming virus (SFFV). F-MuLV behaves as a helper supplying the replication-defective functions to SFFV, while SFFV is the pathogenic component responsible for the acute erythroblastosis. SFFV is responsible for the state of either polycythemia (SFFV_P_) or anemia (SFFV_A_). Further studies have demonstrated that the Rauscher SFFV (Ra-SFFV) was genetically equivalent to the Friend SFFV_A_[34,35]. The conclusive proof that SFFV was the pathogenic component responsible for the acute erythroblastosis was that F-MuLV-free viral preparations containing only the SFFV genome could provoke an erythroid proliferative disease when inoculated in mice [36].

### The role of gp55 in erythroblastosis

The SFFV genome differs from the genome of other acutely oncogenic retroviruses in that it does not contain oncogenic sequences derived from a cellular proto-oncogene. This property is similar to that observed for HTLV-1[37,38]. The pathogenicity of SFFV stems from the product of its *env *gene that encodes a glycoprotein with a molecular mass of 55 kDa (gp55_P _and gp55_A_) [39,40]. The *env *gene contains ecotropic *env *sequences derived from F-MuLV and dual-tropic sequences probably acquired by genetic recombination with *env *sequences from an endogenous polytropic virus present as multiple copies in the mouse genome [41]. Thus, gp55 presents structural elements homologous to polytropic env glycoproteins in its amino-terminal region and elements homologous to the ecotropic MuLV env gp70 in its carboxy-terminal part. The gp55 contains an extracellular domain, a transmembrane domain and a short cytoplasmic tail [42]. Gp55 is not processed in the viral particle but remains in the membranes of the infected cells. A small fraction of gp55 (3–5%) is highly glycosylated in a gp65 form found anchored at the cell surface, while the majority of gp55 remains in the rough endoplasmic reticulum [34]. This glycosylation processing is required for the SFFV leukemogenic activity [43].

To decipher the exact role of gp55 in the Friend disease, various models have been elaborated. A retroviral vector transducing only the gene encoding gp55_P _was constructed. Upon inoculation in mice, it induced the early stage of the Friend disease similarly to that achieved with the entire SFFV_P _genome [44]. Another study used transgenesis. The gp55_P_-transgenic mice developed a polycythemia associated with a massive proliferation of erythroid precursor cells that were not transplantable [45]. Thus, these elegant approaches provided convincing evidence that gp55 is directly responsible for the early erythroblastosis.

An important insight into the gp55 function in erythroid precursor cells was the demonstration that gp55 directly interacts with the receptor for erythropoietin (EpoR) [46]. This interaction occurs through the respective transmembrane domains of EpoR and gp55. The binding of gp55 to EpoR, that is highly expressed at the transition of late BFU-E to CFU-E, results in EpoR activation and promotes the Epo-independent proliferation and differentiation of erythroid progenitor cells [42,43,47,48]. Rare amino acid differences have been identified in the transmembrane domains of gp55_P _and gp55_A_. Due to these differences, the level of EpoR activation remains incomplete with gp55_A _explaining the increase in Epo sensitivity [49] but not autonomy from Epo. In addition to EpoR, gp55 also recruits the short form of the stem-cell-kinase receptor (sf-Stk) as a signaling partner [50,51]. Stk/RON is a tyrosine kinase receptor of the Met family. sf-Stk retains the transmembrane and the tyrosine kinase domains, but lacks the extracellular ligand binding domain of Stk [51-53]. Stk/RON is encoded by the *Fv2 *gene (Friend virus susceptibility gene 2) [52], previously known as a gene determining the susceptibility of some strains of mice (Fv2^s/s^) to the Friend disease [54,55]. Transcription of *sf-Stk *is naturally initiated from an internal promoter within the *Stk *gene, and this internal promoter is deleted in mice resistant to Friend virus (Fv2^r/r^) [52]. Strikingly, the pathology that developed in gp55_P_-transgenic mice was not observed under the genetic *Fv2*^*r*/*r *^background [45], emphasizing the essential role of sf-Stk in gp55-mediated erythroid proliferation. In SFFV-infected mice, gp55, EpoR and sf-Stk are effectors in signaling pathways which encompass the signal transducers and transcriptional activators STATs [56,57], PI3-kinase/AKT [50], the molecular adaptors Grb2/Gab2 [58], the Lyn kinase [59], the p38MAP kinase [60], and the ERK1/2 MAP kinases [61,62]. Constitutive activation of these signaling pathways by the gp55/EpoR and gp55/sf-Stk complexes leads to the dysregulation of proliferation, survival and differentiation of SFFV_P_-infected erythroid progenitors in Fv2^s/s ^mice which result in acute erythroblastosis.

### The oncogenic events during Friend erythroleukemia

Although SFFV is an acutely transforming retrovirus, it induces malignant transformation of erythroid cells by a mechanism of insertional mutagenesis which is usually employed by non-acute leukemogenic virus. Identification of SFFV proviruses integration sites in the genome of various Friend tumor cells was the first molecular proof for the clonal nature of the leukemic transformation [63]. The molecular characterization of the SFFV integration sites revealed that SFFV_P _or SFFV_A _preferentially integrates at the same genomic locus in 95% of the Friend tumor cells, called *spi-1 *for SFFV proviral integration site-1 [64,65]. The *spi-1 *locus is transcriptionally activated by the transcriptional enhancers present in the SFFV LTR [66,67], producing the overexpression of a normal *spi-1 *mRNA translated into a normal Spi-1 protein [66,68]. During DMSO-induced erythroid differentiation of Friend tumor cells, it was noticed that one of the early events was a marked decline in the level of Spi-1 expression [69]. Based on this observation, it was proposed that the arrest in erythroid differentiation was, at least in part, due to Spi-1 overexpression. The high recurrence of *Spi*-1 insertional mutation in Friend tumor cells suggested that Spi-1 overexpression was cooperative with the constitutive signaling from gp55/EpoR and sf-Stk/EpoR complexes to induce the malignant transformation of the proerythroblast. Indeed, in a heterologous avian model of erythroid self-renewal and differentiation, the effects of Spi-1 on proliferation, survival and differentiation arrest required the co-expression of Spi-1 with an EpoR activated either by gp55 or by a mutation on the residue R129C, which mimics EpoR/gp55 activation [70-72].

Besides Spi-1 overexpression, recurrent alterations in the tumor suppressor *p53 *gene were also detected in the late stage of the Friend disease. Allelic deletions or missense mutations led to loss of p53 tumor suppressive function [73-75], and it was shown that Friend leukemia develop more rapidly in transgenic mice expressing a mutant p53 allele, or in p53-null mice, than in normal mice [76,77]. Although the ectopic expression of a normal p53 protein in Friend tumor cells induces apoptosis and hemoglobin production [78], the loss or the mutation of p53 seems to favor the growth and survival of leukemic proerythroblasts rather than specifically altering erythroid differentiation.

### The role of Spi-1 in the arrest of erythroid differentiation

To specify the role of Spi-1 in erythroleukemia, a murine model of *spi-1 *transgenesis has been constructed [79]. The *spi-1 *transgene, placed under the control of the SFFV_P _LTR, was introduced into the germinal lineage of *Fv2*^*s*/*s *^mice. *Spi-1 *transgenic mice develop, within 3 to 4 months after birth, a severe anemia and a massive swelling of the spleen followed by an enlargement of the liver. Bone marrow, spleen, and liver are infiltrated by proerythroblasts arrested in differentiation, mostly at the basophilic stage. These proerythroblastic cells can be established *in vitro *as permanent cell lines that are exquisitely dependent on Epo for proliferation and survival, and the cells are unable to induce tumors when engrafted *in vivo*. The Epo dependency was confirmed *in vivo *by the demonstration that elevation of hematocrit levels (above 60%) by repeated red blood cells transfusions caused regression of the hepatosplenomegaly and disappearance of circulating blasts. Nonetheless, in the animal, erythroblasts expansion occurs in both the presence of Epo in the plasma and the secretion of SCF (stem cell factor) in the marrow or spleen microenvironment. In fact, SCF cooperates with Epo to maintain the *in vitro *survival and proliferation of the *spi-1 *transgenic proerythroblasts when Epo is used at limiting concentrations [80]. Thus, the *spi-1 *transgenic model demonstrates that an ectopic expression of Spi-1 results in a block in the maturation program of erythroid precursor cells without removing their growth factor requirement for survival.

The function of Spi-1 in the erythroid differentiation blockage is multifaceted. The extinction of Spi-1 by *spi-1*-interfering RNAs in *spi-1 *transgenic proerythroblasts is sufficient to reinstate the erythroid differentiation program [81]. This differentiation process is associated with an arrest in proliferation due to both cell death by apoptosis and cell cycle arrest. The mechanisms controlled by Spi-1 that permit survival of the proerythroblast and avoid an exit from the cell cycle are still subjected to speculation. Spi-1 is identical to the transcription factor PU.1, a member of the ETS family. Studies with PU.1 knock-out mice have shown that PU.1 supports hematopoiesis at different stages. Deficiency in PU.1 results in the selective loss of B lymphoid and macrophage development, and a delayed development of neutrophils and T cells [82-86]. In addition, PU.1 plays a role in regulating the commitment of multipotent hematopoietic progenitors [87,88]. Spi-1/PU.1 functions require the ability of the protein to bind purine-rich DNA sequences in the promoters and enhancers of target genes [68,89]. Distinct threshold levels of PU.1 also determine function, with high levels driving precursors to a myeloid cell fate, while moderate levels specify B lymphoid development [90]. Notably, Spi-1/PU.1 down-regulation is required for normal erythroid development [91,92]. Thus, Spi-1 overexpression in the proerythroblast probably changes the delicate balance of transcriptional activities required for normal erythropoiesis.

It was postulated that excess Spi-1 may disrupt the function of an erythroid factor and, in this hypothesis, GATA-1 was an obvious candidate [93-95]. Indeed, GATA-1-deficient erythroid cells fail to mature beyond the proerythroblast stage and die by apoptosis [96,97]. Several studies have reported a reciprocal inhibition of Spi-1/PU.1 and GATA-1 functions through direct interaction [98-101]. Furthermore, the concept that Spi-1 could inhibit the function of GATA-1 in erythroleukemic cells is supported by the reversal of tumorigenicity and the re-initiation of a differentiation program when GATA-1 expression is ectopically imposed in a Friend tumor cell line [102]. Nonetheless, target genes for GATA-1, such as EKLF, NF-E2, β globin, EpoR and GATA-1-itself are expressed in both Friend and *spi-1 *transgenic cells [79](our unpublished data) indicating that some GATA-1 functions are not abolished by Spi-1 overexpression. Global transcriptome analyses of preleukemic *spi-1 *transgenic proerythroblasts should provide data allowing the elucidation of the direct targets downstream Spi-1 and the molecular pathways leading to erythroleukemogenesis. As a first indication, transcription of the *fli-1 *gene is directly regulated by Spi-1 in Friend tumor cells [103]. Fli-1 is a transcription factor of the ETS family that behaves as an oncogene when activated by insertional mutagenesis in late erythroid tumors induced by the Friend MuLV alone [104]. Thus, Fli-1 dysregulation may contribute, in part, to the erythroid differentiation arrest induced by Spi-1. Another mechanism may involve the phosphatase SHP-1 [105] that is transcriptionally up regulated by Spi-1 [106]. SHP-1 may interfere with the activation of STAT1 and STAT3 and inhibit their DNA binding activities, contributing to the erythroid differentiation arrest[57].

### The two-stage erythroleukemia in *spi-1 *transgenic mice

**Figure 2:**
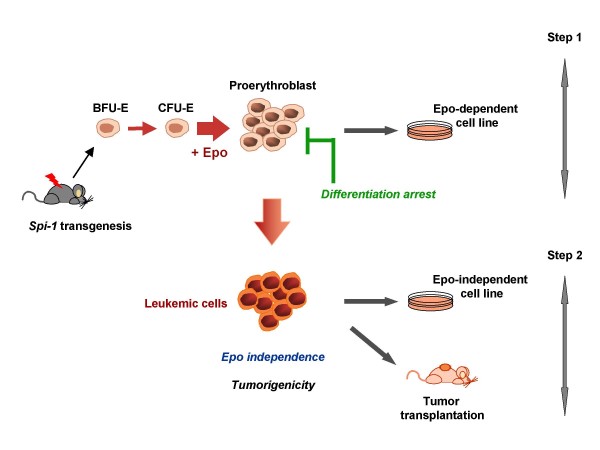
**Erythroleukemia in *spi-1 *transgenic mice is a two-step disease**. The first step occurring in *spi-1 *transgenic mice is a massive proliferation of proerythroblasts, arrested in differentiation around the CFU-E/proerythroblast stage. These cells can be established *in vitro *as Epo-dependent cell lines. In a second step, malignant proerythroblastic cells emerge that proliferate *in vitro *without Epo and are tumorigenic when grafted into Nude mice.

During disease progression, fully transformed proerythroblastic cells emerge that are characterized by both autonomous growth *in vitro *and tumorigenicity *in vivo*. Thus, like the disease induced by the Friend virus complex, *spi-1 *transgenic mice develop a malignant process that evolves in two steps (Figure 2) whereby the blastic phase reflects the outgrowth of a malignant erythroblastic cell subpopulation with acquired genetic lesions. According to the data accumulated with studies on the Friend disease, abnormalities occurring in the *p53 *gene were examined. Inactivating p53 mutations were frequently observed in malignant *spi-1 *transgenic proerythroblasts, and we reported that disease progression was highly accelerated in a *p53*-null background [107]. Nevertheless, Epo-dependent and non-tumorigenic cells can be isolated during the early erythroblastic phase of diseased p53^-/-^-*spi-1 *transgenic mice indicating that *p53 *germline deletion is not sufficient to confer the malignant phenotype in this context [108]. As for the Friend disease, *p53 *abnormalities appear more as a permissive event supporting the illegitimate survival of proerythroblasts harboring genetic aberrations than a direct transformation event.

**Figure 3:**
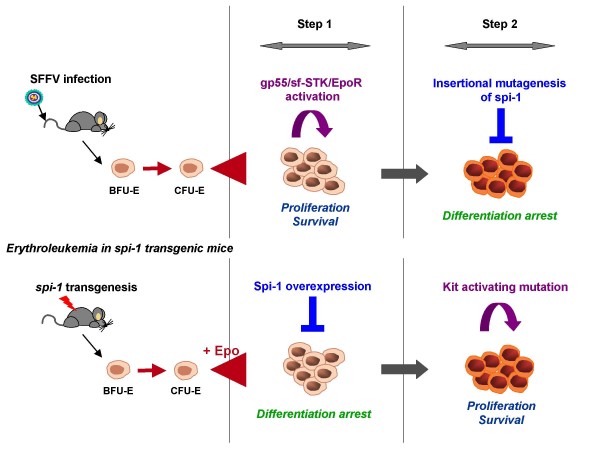
**Oncogenic cooperation in murine erythroleukemia**. During Friend acute erythroleukemia, the early expansion of erythroid progenitors is due to the activation of EpoR and sf-STK by the viral gp55. The blastic crisis is associated with the overexpression of the transcription factor Spi-1/PU.1, occurring by insertional mutagenesis due to SFFV. During erythroleukemia progression in *spi-1 *transgenic mice, the initial oncogenic event is the overexpression of Spi-1/PU.1 by germinal mutation that induces a differentiation arrest of the proerythroblast. The blastic crisis is associated with activating mutations in the *Kit *gene that promote the Epo-independent proliferation and survival of the malignant proerythroblast.

Next, signaling alterations were prospected. These studies led to the identification of point mutations in the *Kit *gene in 86% of tumors isolated late during leukemia progression [80]. Kit, the tyrosine kinase receptor for SCF, is expressed on hematopoietic stem cells and committed progenitor cells of the different blood cell lineages [109,110]. Kit is activated by SCF binding, which induces receptor dimerization followed by the activation of the intrinsic tyrosine kinase and receptor transphosphorylation on specific tyrosine residues [111,112]. By triggering multiple signaling pathways including ERK1/2 MAP kinases, PI3Kinase, and Src kinases, Kit activation modulates cell survival and proliferation [113-115]. Most Kit mutations found in *spi-1 *transgenic leukemic cells affect amino acids located in the Kit catalytic domain (mainly codon 814 and occasionally codon 818). Of note, similar mutations were found in human mastocytosis [116,117] and acute myeloid leukemia [118-120]. These gain-of-function mutations confer ligand-independent tyrosine kinase activity to Kit. The mutated forms of Kit constitutively activate MAP kinases and PI3Kinase/AKT pathways in the *spi-1 *transgenic leukemic cells [80,121]. When the expression of Kit mutants is enforced in *spi-1 *transgenic preleukemic proerythroblasts, cells become growth-factor independent and tumorigenic *in vivo*. Thus, a constitutive signaling from Kit mutants combined to Spi-1 overexpression leads to malignant transformation of proerythroblasts (Figure 3).

### From murine to human

AML in human are characterized by an uncontrolled expansion of immature blasts, which fail to differentiate normally. These leukemias are classified into sub-types according to the predominance of the altered myeloid lineage. It is currently accepted that the disease progresses from a chronic phase to an acute blastic crisis. Molecularly, leukemias are associated with multiple genetic alterations, including chromosomal translocations and mutations. Overviews on the genetic alterations found in AML are detailed in recent reviews [122-127]. One important observation is that the genetic alterations can be divided into two classes. One class targets transcription factors that play a regulatory role in hematopoietic development. These transcription factors are frequently modified through chromosomal translocations and the resulting chimeric proteins inhibit differentiation in a particular hematopoietic cell lineage. For example, the promyelocytic leukemia-retinoic acid receptor α (PML/RARα) expressed from the t(15;17) translocation is associated with 99% of acute promyelocytic leukemia [128]. PML/RARα behaves as a transcriptional repressor for the hormonal receptor RARα [129]. In the second class, mutations affect signaling effectors like tyrosine kinases [125]. These mutations are gain-of-function and provide proliferative signals, as exemplified by point mutations inducing the constitutive activation of the tyrosine kinase receptor FLT3 in ~30% of AML [122,130,131]. Strikingly, murine models designed to express one genetically modified protein, using the transplantation of retrovirally-transduced bone marrow cell or transgenesis, showed that one type of mutation does not induce an acute leukemia on its own. Generally, they provoke a myeloproliferative disorder. Progression to leukemia requires either a long latency, making plausible the occurrence of a second mutation, or an additional treatment with mutagenic compounds. The concept emerging from this complexity is that an acute leukemia arises from the cooperation between two events: one mutation interfering with differentiation and the other mutation conferring a proliferation advantage [132].

Both the Friend and *spi-1 *transgenic models demonstrate that leukemia development depends on the cooperation between one mutation that impairs differentiation and a second mutation that promotes autonomous cell growth (Figure 1). In that way, these murine models provide proof-of-principle for the "two-hits" model of leukemogenesis [132]. They highlight that complementation between the two classes of mutations is more important for leukemogenesis than the timing order of their occurrence.

## Conclusion

Significant advances have been made in the mechanisms of leukemia from studies on murine models of erythroleukemia. In the nineteen-sixties, these studies established the concept of multiple-step evolution of leukemia. Forty years later, these models validate the concept of oncogenic cooperativity merging the diversity of genetic lesions that underlie the development of acute myeloid leukemia in human. A future challenge will be to integrate the networks of transcriptional regulation and signaling toward a better understanding of leukemogenic processes.

## Competing interests

The author(s) declare that they have no competing interests.

## References

[B1] Kabat D (1989). Molecular biology of Friend viral erythroleukemia. Curr Top Microbiol Immunol.

[B2] Chesebro B, Miyazawa M, Britt WJ (1990). Host genetic control of spontaneous and induced immunity to Friend murine retrovirus infection. Annu Rev Immunol.

[B3] Goff SP (2004). Retrovirus restriction factors. Mol Cell.

[B4] Miyazawa M, Tsuji-Kawahara S, Kanari Y (2008). Host genetic factors that control immune responses to retrovirus infections. Vaccine.

[B5] Lilly F (1967). Susceptibility to two strains of Friend leukemia virus in mice. Science.

[B6] Pincus T, Rowe WP, Lilly F (1971). A major genetic locus affecting resistance to infection with murine leukemia viruses. II. Apparent identity to a major locus described for resistance to friend murine leukemia virus. J Exp Med.

[B7] Jolicoeur P (1979). The Fv-1 gene of the mouse and its control of murine leukemia virus replication. Curr Top Microbiol Immunol.

[B8] Best S, Le Tissier P, Towers G, Stoye JP (1996). Positional cloning of the mouse retrovirus restriction gene Fv1. Nature.

[B9] Towers G, Bock M, Martin S, Takeuchi Y, Stoye JP, Danos O (2000). A conserved mechanism of retrovirus restriction in mammals. Proc Natl Acad Sci USA.

[B10] Stremlau M, Owens CM, Perron MJ, Kiessling M, Autissier P, Sodroski J (2004). The cytoplasmic body component TRIM5alpha restricts HIV-1 infection in Old World monkeys. Nature.

[B11] Hatziioannou T, Perez-Caballero D, Yang A, Cowan S, Bieniasz PD (2004). Retrovirus resistance factors Ref1 and Lv1 are species-specific variants of TRIM5alpha. Proc Natl Acad Sci USA.

[B12] Stevens A, Bock M, Ellis S, LeTissier P, Bishop KN, Yap MW, Taylor W, Stoye JP (2004). Retroviral capsid determinants of Fv1 NB and NR tropism. J Virol.

[B13] Lassaux A, Sitbon M, Battini JL (2005). Residues in the murine leukemia virus capsid that differentially govern resistance to mouse Fv1 and human Ref1 restrictions. J Virol.

[B14] Chesebro B, Wehrly K (1979). Identification of a non-H-2 gene (Rfv-3) influencing recovery from viremia and leukemia induced by Friend virus complex. Proc Natl Acad Sci USA.

[B15] Doig D, Chesebro B (1979). Anti-Friend virus antibody is associated with recovery from viremia and loss of viral leukemia cell-surface antigens in leukemic mice. Identification of Rfv-3 as a gene locus influencing antibody production. J Exp Med.

[B16] Stopak K, de Noronha C, Yonemoto W, Greene WC (2003). HIV-1 Vif blocks the antiviral activity of APOBEC3G by impairing both its translation and intracellular stability. Mol Cell.

[B17] Marin M, Rose KM, Kozak SL, Kabat D (2003). HIV-1 Vif protein binds the editing enzyme APOBEC3G and induces its degradation. Nat Med.

[B18] Santiago ML, Montano M, Benitez R, Messer RJ, Yonemoto W, Chesebro B, Hasenkrug KJ, Greene WC (2008). Apobec3 encodes Rfv3, a gene influencing neutralizing antibody control of retrovirus infection. Science.

[B19] Takeda E, Tsuji-Kawahara S, Sakamoto M, Langlois MA, Neuberger MS, Rada C, Miyazawa M (2008). Mouse APOBEC3 restricts Friend leukemia virus infection and pathogenesis in vivo. J Virol.

[B20] Friend C (1957). Cell-free transmission in adult Swiss mice of a disease having the character of a leukemia. J Exp Med.

[B21] Tambourin PE, Wendling F, Jasmin C, Smadja-Joffe F (1979). The physiopathology of Friend leukemia. Leuk Res.

[B22] Rauscher FJ (1962). A virus-induced disease of mice characterized by erythrocytopoiesis and lymphoid leukemia. J Natl Cancer Inst.

[B23] Mirand EA (1967). Virus-induced erythropoiesis in hypertransfused-polycythemic mice. Science.

[B24] Mirand EA, Steeves RA, Lange RD, Grace JT (1968). Virus-induced polycythemia in mice: erythropoiesis without erythropoietin. Proc Soc Exp Biol Med.

[B25] Tambourin P, Wendling F (1971). Malignant transformation and erythroid differentiation by polycythaemia-inducing Friend virus. Nat New Biol.

[B26] Liao SK, Axelrad AA (1975). Erythropoietin-independent erythroid colony formation in vitro by hemopoietic cells of mice infected with friend virus. Int J Cancer.

[B27] Tambourin PE, Wendling F (1975). Target cell for oncogenic action of polycythaemia-inducing Friend virus. Nature.

[B28] Fredrickson T, Tambourin P, Wendling F, Jasmin C, Smajda F (1975). Target cell of the polycythemia-inducing Friend virus: studies with myleran. J Natl Cancer Inst.

[B29] Tambourin P, Wendling F, Moreau-Gachelin F (1981). Friend leukemia as a multiple-step disease. Blood Cells.

[B30] Mager DL, Mak TW, Bernstein A (1981). Quantitative colony method for tumorigenic cells transformed by two distincts strains of Friend leukemia virus. Proc Nat Acad Sci USA.

[B31] Ben-David Y, Bernstein A (1991). Friend virus-induced erythroleukemia and the multistage nature of cancer. Cell.

[B32] Marks PA, Rifkind RA (1978). Erythroleukemic differentiation. Annu Rev Biochem.

[B33] Gillet N, Florins A, Boxus M, Burteau C, Nigro A, Vandermeers F, Balon H, Bouzar AB, Defoiche J, Burny A, Reichert M, Kettmann R, Willems L (2007). Mechanisms of leukemogenesis induced by bovine leukemia virus: prospects for novel anti-retroviral therapies in human. Retrovirology.

[B34] Ruta M, Kabat D (1980). Plasma membrane glycoproteins encoded by cloned Rauscher and Friend spleen focus-forming viruses. J Virol.

[B35] Vogt M, Haggblom C, Swift S, Haas M (1985). Envelope gene and long terminal repeat determine the different biological properties of Rauscher, Friend, and Moloney mink cell focus-inducing viruses. J Virol.

[B36] Wolff L, Ruscetti S (1985). Malignant transformation of erythroid cells in vivo by introduction of a nonreplicating retrovirus vector. Science.

[B37] Matsuoka M, Jeang KT (2007). Human T-cell leukaemia virus type 1 (HTLV-1) infectivity and cellular transformation. Nat Rev Cancer.

[B38] Matsuoka M (2005). Human T-cell leukemia virus type I (HTLV-I) infection and the onset of adult T-cell leukemia (ATL). Retrovirology.

[B39] Linemeyer DL, Menke JG, Ruscetti SK, Evans LH, Scolnick EM (1982). Envelope gene sequences which encode the gp52 protein of spleen focus-forming virus are required for the induction of erythroid cell proliferation. J Virol.

[B40] Ruta M, Bestwick R, Machida C, Kabat D (1983). Loss of leukemogenicity caused by mutations in the membrane glycoprotein structural gene of Friend spleen focus-forming virus. Proc Natl Acad Sci USA.

[B41] Clark SP, Mak TW (1984). Fluidity of a retrovirus genome. Journal of Virology.

[B42] Zon LI, Moreau JF, Koo JW, Mathey-Prevot B, D'Andrea AD (1992). The erythropoietin receptor transmembrane region is necessary for activation by the Friend spleen focus-forming virus gp55 glycoprotein. Mol Cell Biol.

[B43] Li JP, Hu HO, Niu QT, Fang C (1995). Cell surface activation of the erythropoietin receptor by friend spleen focus-forming virus gp55. J Virol.

[B44] Wolff L, Ruscetti S (1988). The spleen focus-forming virus (SFFV) envelope gene, when introduced into mice in the absence of other SFFV genes, induces acute erythroleukemia. J Virol.

[B45] Aizawa S, Suda Y, Furuta Y, Yagi T, Takeda N, Watanabe N, Nagayoshi M, Ikawa Y (1990). *Env*-derived gp55 gene of Friend spleen focus-forming virus specifically induces neoplastic proliferation of erythroid progenitor cells. EMBO J.

[B46] Li JP, D'Andrea AD, Lodish HF, Baltimore D (1990). Activation of cell growth by binding of Friend spleen focus-forming virus gp55 glycoprotein to the erythropoietin receptor. Nature.

[B47] Chung SW, Wolff L, Ruscetti SK (1989). Transmembrane domain of the envelope gene of a polycythemia-inducing retrovirus determines erythropoietin-independent growth. Proc Natl Acad Sci USA.

[B48] Ruscetti SK, Janesch NJ, Chakraborti A, Sawyer ST, Hankins WD (1990). Friend Spleen-Focus-forming virus induces factor independence in an erythropoietin-dependent erythroleukemia cell line. J Virol.

[B49] Constantinescu SN, Keren T, Russ WP, Ubarretxena-Belandia I, Malka Y, Kubatzky KF, Engelman DM, Lodish HF, Henis YI (2003). The erythropoietin receptor transmembrane domain mediates complex formation with viral anemic and polycythemic gp55 proteins. J Biol Chem.

[B50] Nishigaki K, Thompson D, Hanson C, Yugawa T, Ruscetti S (2001). The envelope glycoprotein of friend spleen focus-forming virus covalently interacts with and constitutively activates a truncated form of the receptor tyrosine kinase Stk. J Virol.

[B51] Zhang J, Randall MS, Loyd MR, Li W, Schweers RL, Persons DA, Rehg JE, Noguchi CT, Ihle JN, Ney PA (2006). Role of erythropoietin receptor signaling in Friend virus-induced erythroblastosis and polycythemia. Blood.

[B52] Persons DA, Paulson RF, Loyd MR, Herley MT, Bodner SM, Bernstein A, Correll PH, Ney PA (1999). Fv2 encodes a truncated form of the Stk receptor tyrosine kinase. Nat Genet.

[B53] Rulli K, Yugawa T, Hanson C, Thompson D, Ruscetti S, Nishigaki K (2004). Ex vivo and in vivo biological effects of a truncated form of the receptor tyrosine kinase stk when activated by interaction with the friend spleen focus-forming virus envelope glycoprotein or by point mutation. J Virol.

[B54] Lilly F (1970). Fv-2: identification and location of a second gene governing the spleen focus response to Friend leukemia virus in mice. J Natl Cancer Inst.

[B55] Iwama A, Okano K, Sudo T, Matsuda Y, Suda T (1994). Molecular cloning of a novel receptor tyrosine kinase gene, STK, derived from enriched hematopoietic stem cells. Blood.

[B56] Yamamura Y, Senda H, Kageyama Y, Matsuzaki T, Noda M, Ikawa Y (1998). Erythropoietin and friend virus gp55 activate different JAK/STAT pathways through the erythropoietin receptor in erythroid cells. Mol Cell Biol.

[B57] Nishigaki K, Hanson C, Ohashi T, Spadaccini A, Ruscetti S (2006). Erythroblast transformation by the friend spleen focus-forming virus is associated with a block in erythropoietin-induced STAT1 phosphorylation and DNA binding and correlates with high expression of the hematopoietic phosphatase SHP-1. J Virol.

[B58] Teal HE, Ni S, Xu J, Finkelstein LD, Cheng AM, Paulson RF, Feng GS, Correll PH (2006). GRB2-mediated recruitment of GAB2, but not GAB1, to SF-STK supports the expansion of Friend virus-infected erythroid progenitor cells. Oncogene.

[B59] Subramanian A, Hegde S, Correll PH, Paulson RF (2006). Mutation of the Lyn tyrosine kinase delays the progression of Friend virus induced erythroleukemia without affecting susceptibility. Leuk Res.

[B60] Jelacic TM, Thompson D, Hanson C, Cmarik JL, Nishigaki K, Ruscetti S (2008). The tyrosine kinase sf-Stk and its downstream signals are required for maintenance of friend spleen focus-forming virus-induced fibroblast transformation. J Virol.

[B61] Muszynski KW, Ohashi T, Hanson C, Ruscetti SK (1998). Both the polycythemia- and anemia-inducing strains of Friend spleen focus-forming virus induce constitutive activation of the Raf-1/mitogen-activated protein kinase signal transduction pathway. J Virol.

[B62] Muszynski KW, Thompson D, Hanson C, Lyons R, Spadaccini A, Ruscetti SK (2000). Growth factor-independent proliferation of erythroid cells infected with friend spleen focus-forming virus is protein kinase C dependent but does not require ras-GTP [In Process Citation]. J Virol.

[B63] Moreau-Gachelin F, Robert-Lezenes J, Wendling F, Tavitian A, Tambourin P (1985). Integration of spleen focus-forming virus proviruses in Friend tumor cells. J Virol.

[B64] Moreau-Gachelin F, Tavitian A, Tambourin P (1988). *Spi-1 *is a putative oncogene in virally induced murine erythroleukemia. Nature (London).

[B65] Paul R, Schuetze S, Kozak SL, Kabat D (1989). A common site for immortalizing proviral integrations in Friend erythroleukemia: molecular cloning and characterization. J Virol.

[B66] Moreau-Gachelin F, Ray D, Mattei MG, Tambourin P, Tavitian A (1989). The putative oncogene Spi-1: murine chromosomal localization and transcriptional activation in murine acute erythroleukemias [published erratum appears in Oncogene 1990 Jun;5(6):941]. Oncogene.

[B67] Okuno Y, Huang G, Rosenbauer F, Evans EK, Radomska HS, Iwasaki H, Akashi K, Moreau-Gachelin F, Li Y, Zhang P, Göttgens B, Tenen DG (2005). Potential autoregulation of transcription factor PU.1 by an upstream regulatory element. Mol Cell Biol.

[B68] Paul R, Schuetze S, Kozak SL, Kozak CA, Kabat D (1991). The Sfpi-1 proviral integration site of Friend erythroleukemia encodes the ets-related transcription factor Pu.1. J Virol.

[B69] Schuetze S, Paul R, Gliniak BC, Kabat D (1992). Role of the PU.1 transcription factor in controlling differentiation of Friend erythroleukemia cells. Mol Cell Biol.

[B70] Longmore GD, Lodish HF (1991). An activating mutation in the murine erythropoietin receptor induces erythroleukemia in mice: a cytokine receptor superfamily oncogene. Cell.

[B71] Tran Quang C, Wessely O, Pironin M, Beug H, Ghysdael J (1997). Cooperation of Spi-1/PU.1 with an activated erythropoietin receptor inhibits apoptosis and Epo-depedent differenciation in primary erythroblasts and induces their kit ligand-dependent proliferation. EMBO J.

[B72] Pereira R, Raingeaud J, Pironin M, Ghysdael J, Quang CT (2000). SPI-1 transforming properties depend upon specifically activated forms of the EPOR. Oncogene.

[B73] Mowat M, Cheng A, Kimura N, Bernstein A, Benchimol S (1985). Rearrangements of the cellular p53 gene in erythroleukaemic cells transformed by Friend virus. Nature.

[B74] Ben-David Y, Prideaux VR, Chow V, Benchimol SaBA (1988). Inactivation of the p53 oncogene by internal deletion or retroviral integration in erythroleukemic cell lines induced by Friend leukemia virus. Oncogene.

[B75] Munroe DG, Peacock JW, Benchimol S (1990). Inactivation of the cellular p53 gene is a common feature of Friend virus-induced erythroleukemia: relationship of inactivation to dominant transforming alleles. Mol Cell Biol.

[B76] Lavigueur A, Bernstein A (1991). p53 transgenic mice: accelerated erythroleukemia induction by Friend virus. Oncogene.

[B77] Prasher JM, Elenitoba-Johnson KS, Kelley LL (2001). Loss of p53 tumor suppressor function is required for in vivo progression of Friend erythroleukemia. Oncogene.

[B78] Johnson P, Chung S, Benchimol S (1993). Growth suppression of Friend virus-transformed erythroleukemia cells by p53 protein is accompanied by hemoglobin production and is sensitive to erythropoietin. Mol Cell Biol.

[B79] Moreau-Gachelin F, Wendling F, Molina T, Denis N, Titeux M, Grimber G, Briand P, Vainchenker W, Tavitian A (1996). Spi-1/PU.1 transgenic mice develop multistep erythroleukemias. Mol Cell Biol.

[B80] Kosmider O, Denis N, Lacout C, Vainchenker W, Dubreuil P, Moreau-Gachelin F (2005). Kit-activating mutations cooperate with Spi-1/PU.1 overexpression to promote tumorigenic progression during erythroleukemia in mice. Cancer Cell.

[B81] Rimmele P, Kosmider O, Mayeux P, Moreau-Gachelin F, Guillouf C (2007). Spi-1/PU.1 participates in erythroleukemogenesis by inhibiting apoptosis in cooperation with Epo signaling and by blocking erythroid differentiation. Blood.

[B82] McKercher SR, Torbett BE, Anderson KL, Henkel GW, Vestal DJ, Baribault H, Klemsz M, Feeney AJ, Wu GE, Paige CJ, Maki RA (1996). Targeted disruption of the PU.1 gene results in multiple hematopoietic abnormalities. Embo J.

[B83] Scott EW, Fisher RC, Olson MC, Kehrli EW, Simon MC, Singh H (1997). PU.1 functions in a cell-autonomous manner to control the differentiation of multipotential lymphoid-myeloid progenitors. Immunity.

[B84] DeKoter RP, Walsh JC, Singh H (1998). PU.1 regulates both cytokine-dependent proliferation and differentiation of granulocyte/macrophage progenitors. Embo J.

[B85] Anderson MK, Weiss AH, Hernandez-Hoyos G, Dionne CJ, Rothenberg EV (2002). Constitutive expression of PU.1 in fetal hematopoietic progenitors blocks T cell development at the pro-T cell stage. Immunity.

[B86] Zou GM, Chen JJ, Yoder MC, Wu W, Rowley JD (2005). Knockdown of Pu.1 by small interfering RNA in CD34+ embryoid body cells derived from mouse ES cells turns cell fate determination to pro-B cells. Proc Natl Acad Sci USA.

[B87] Iwasaki H, Somoza C, Shigematsu H, Duprez EA, Iwasaki-Arai J, Mizuno S, Arinobu Y, Geary K, Zhang P, Dayaram T, Fenyus ML, Elf S, Chan S, Kastner P, Huettner CS, Murray R, Tenen DG, Akashi K (2005). Distinctive and indispensable roles of PU.1 in maintenance of hematopoietic stem cells and their differentiation. Blood.

[B88] Dakic A, Metcalf D, Di Rago L, Mifsud S, Wu L, Nutt SL (2005). PU.1 regulates the commitment of adult hematopoietic progenitors and restricts granulopoiesis. J Exp Med.

[B89] Klemsz MJ, McKercher SR, Celada A, Van Beveren C, Maki RA (1990). The macrophage and B cell-specific transcription factor PU.1 is related to the ets oncogene. Cell.

[B90] DeKoter RP, Singh H (2000). Regulation of B lymphocyte and macrophage development by graded expression of PU.1. Science.

[B91] Back J, Dierich A, Bronn C, Kastner P, Chan S (2004). PU.1 determines the self-renewal capacity of erythroid progenitor cells. Blood.

[B92] Nutt SL, Metcalf D, D'Amico A, Polli M, Wu L (2005). Dynamic regulation of PU.1 expression in multipotent hematopoietic progenitors. J Exp Med.

[B93] Tsai SF, Martin DI, Zon LI, D'Andrea AD, Wong GG, Orkin SH (1989). Cloning of cDNA for the major DNA-binding protein of the erythroid lineage through expression in mammalian cells. Nature.

[B94] Evans T, Felsenfeld G (1989). The erythroid-specific transcription factor Eryf1: a new finger protein. Cell.

[B95] Ferreira R, Ohneda K, Yamamoto M, Philipsen S (2005). GATA1 function, a paradigm for transcription factors in hematopoiesis. Mol Cell Biol.

[B96] Pevny L, Simon MC, Robertson E, Klein WH, Tsai SF, D'Agati V, Orkin SH, Costantini F (1991). Erythroid differentiation in chimaeric mice blocked by a targeted mutation in the gene for transcription factor GATA-1. Nature.

[B97] Weiss MJ, Orkin SH (1995). Transcription factor GATA-1 permits survival and maturation of erythroid precursors by preventing apoptosis. Proc Natl Acad Sci USA.

[B98] Rekhtman N, Radparvar F, Evans T, Skoultchi AI (1999). Direct interaction of hematopoietic transcription factors PU.1 and GATA-1: functional antagonism in erythroid cells. Genes Dev.

[B99] Zhang P, Behre G, Pan J, Iwama A, Wara-Aswapati N, Radomska HS, Auron PE, Tenen DG, Sun Z (1999). Negative cross-talk between hematopoietic regulators: GATA proteins repress PU.1. Proc Natl Acad Sci USA.

[B100] Nerlov C, Querfurth E, Kulessa H, Graf T (2000). GATA-1 interacts with the myeloid PU.1 transcription factor and represses PU.1-dependent transcription. Blood.

[B101] Stopka T, Amanatullah DF, Papetti M, Skoultchi AI (2005). PU.1 inhibits the erythroid program by binding to GATA-1 on DNA and creating a repressive chromatin structure. Embo J.

[B102] Choe KS, Radparvar F, Matushansky I, Rekhtman N, Han X, Skoultchi AI (2003). Reversal of tumorigenicity and the block to differentiation in erythroleukemia cells by GATA-1. Cancer Res.

[B103] Starck J, Doubeikovski A, Sarrazin S, Gonnet C, Rao G, Skoultchi A, Godet J, Dusanter-Fourt I, Morle F (1999). Spi-1/PU.1 is a positive regulator of the Fli-1 gene involved in inhibition of erythroid differentiation in friend erythroleukemic cell lines. Mol Cell Biol.

[B104] Ben-David Y, Giddens EB, Letwin K, Bernstein A (1991). Erythroleukemia induction by Friend murine leukemia virus: insertional activation of a new member of the ets gene family, Fli-1, closely linked to c-ets-1. Genes Dev.

[B105] Tsui HW, Siminovitch KA, de Souza L, Tsui FW (1993). Motheaten and viable motheaten mice have mutations in the haematopoietic cell phosphatase gene. Nat Genet.

[B106] Wlodarski P, Zhang Q, Liu X, Kasprzycka M, Marzec M, Wasik MA (2007). PU.1 activates transcription of SHP-1 gene in hematopoietic cells. J Biol Chem.

[B107] Barnache S, Wendling F, Lacombe C, Denis N, Titeux M, Vainchenker W, Moreau-Gachelin F (1998). Spi-1 transgenic mice develop a clonal erythroleukemia which does not depend on p53 mutation. Oncogene.

[B108] Scolan EL, Wendling F, Barnache S, Denis N, Tulliez M, Vainchenker W, Moreau-Gachelin F (2001). Germ-line deletion of p53 reveals a multistage tumor progression in spi-1/PU.1 transgenic proerythroblasts. Oncogene.

[B109] Lennartsson J, Jelacic T, Linnekin D, Shivakrupa R (2005). Normal and oncogenic forms of the receptor tyrosine kinase kit. Stem Cells.

[B110] Munugalavadla V, Kapur R (2005). Role of c-Kit and erythropoietin receptor in erythropoiesis. Crit Rev Oncol Hematol.

[B111] Rottapel R, Reedijk M, Williams DE, Lyman SD, Anderson DM, Pawson T, Bernstein A (1991). The Steel/W transduction pathway: kit autophosphorylation and its association with a unique subset of cytoplasmic signaling proteins is induced by the Steel factor. Mol Cell Biol.

[B112] Reith AD, Ellis C, Lyman SD, Anderson DM, Williams DE, Bernstein A, Pawson T (1991). Signal transduction by normal isoforms and W mutant variants of the Kit receptor tyrosine kinase. Embo J.

[B113] Serve H, Yee NS, Stella G, Sepp-Lorenzino L, Tan JC, Besmer P (1995). Differential roles of PI3-kinase and Kit tyrosine 821 in Kit receptor-mediated proliferation, survival and cell adhesion in mast cells. Embo J.

[B114] Timokhina I, Kissel H, Stella G, Besmer P (1998). Kit signaling through PI 3-kinase and Src kinase pathways: an essential role for Rac1 and JNK activation in mast cell proliferation. Embo J.

[B115] Wandzioch E, Edling CE, Palmer RH, Carlsson L, Hallberg B (2004). Activation of the MAP kinase pathway by c-Kit is PI-3 kinase dependent in hematopoietic progenitor/stem cell lines. Blood.

[B116] Longley BJ, Tyrrell L, Lu SZ, Ma YS, Langley K, Ding TG, Duffy T, Jacobs P, Tang LH, Modlin I (1996). Somatic c-KIT activating mutation in urticaria pigmentosa and aggressive mastocytosis: establishment of clonality in a human mast cell neoplasm. Nat Genet.

[B117] Pignon JM, Giraudier S, Duquesnoy P, Jouault H, Imbert M, Vainchenker W, Vernant JP, Tulliez M (1997). A new c-kit mutation in a case of aggressive mast cell disease. Br J Haematol.

[B118] Gari M, Goodeve A, Wilson G, Winship P, Langabeer S, Linch D, Vandenberghe E, Peake I, Reilly J (1999). c-kit proto-oncogene exon 8 in-frame deletion plus insertion mutations in acute myeloid leukaemia. Br J Haematol.

[B119] Beghini A, Peterlongo P, Ripamonti CB, Larizza L, Cairoli R, Morra E, Mecucci C (2000). C-kit mutations in core binding factor leukemias. Blood.

[B120] Wang YY, Zhou GB, Yin T, Chen B, Shi JY, Liang WX, Jin XL, You JH, Yang G, Shen ZX, Chen J, Xiong SM, Chen GQ, Xu F, Liu YW, Chen Z, Chen SJ (2005). AML1-ETO and C-KIT mutation/overexpression in t(8;21) leukemia: Implication in stepwise leukemogenesis and response to Gleevec. Proc Natl Acad Sci U S A.

[B121] Barnache S, Mayeux P, Payrastre B, Moreau-Gachelin F (2001). Alterations of the phosphoinositide 3-kinase and mitogen-activated protein kinase signaling pathways in the erythropoietin-independent Spi-1/PU.1 transgenic proerythroblasts. Blood.

[B122] Gilliland DG, Griffin JD (2002). The roles of FLT3 in hematopoiesis and leukemia. Blood.

[B123] Speck NA, Gilliland DG (2002). Core-binding factors in haematopoiesis and leukaemia. Nat Rev Cancer.

[B124] Tenen DG (2003). Disruption of differentiation in human cancer: AML shows the way. Nat Rev Cancer.

[B125] Reilly JT (2003). Receptor tyrosine kinases in normal and malignant haematopoiesis. Blood Rev.

[B126] Reilly JT (2005). Pathogenesis of acute myeloid leukaemia and inv(16)(p13;q22): a paradigm for understanding leukaemogenesis?. Br J Haematol.

[B127] Rosenbauer F, Koschmieder S, Steidl U, Tenen DG (2005). Effect of transcription-factor concentrations on leukemic stem cells. Blood.

[B128] Scolnik MP, Palacios MF, Acevedo SH, Castuma MV, Larripa IB, Palumbo A, Moiraghi EB, Sasot AM, Huberman AB (1998). Promyelocytic blast crisis of chronic myelogenous leukaemia with translocations (9;22) and (15;17). Leuk Lymphoma.

[B129] Melnick A, Licht JD (1999). Deconstructing a disease: RARalpha, its fusion partners, and their roles in the pathogenesis of acute promyelocytic leukemia. Blood.

[B130] Yamamoto Y, Kiyoi H, Nakano Y, Suzuki R, Kodera Y, Miyawaki S, Asou N, Kuriyama K, Yagasaki F, Shimazaki C, Akiyama H, Saito K, Nishimura M, Motoji T, Shinagawa K, Takeshita A, Saito H, Ueda R, Ohno R, Naoe T (2001). Activating mutation of D835 within the activation loop of FLT3 in human hematologic malignancies. Blood.

[B131] Kottaridis PD, Gale RE, Frew ME, Harrison G, Langabeer SE, Belton AA, Walker H, Wheatley K, Bowen DT, Burnett AK, Goldstone AH, Linch DC (2001). The presence of a FLT3 internal tandem duplication in patients with acute myeloid leukemia (AML) adds important prognostic information to cytogenetic risk group and response to the first cycle of chemotherapy: analysis of 854 patients from the United Kingdom Medical Research Council AML 10 and 12 trials. Blood.

[B132] Gilliland DG (2001). Hematologic malignancies. Curr Opin Hematol.

